# Immunological and Clinical Responses following the Use of Antiretroviral Therapy among Elderly HIV-Infected Individuals Attending Care and Treatment Clinic in Northwestern Tanzania: A Retrospective Cohort Study

**DOI:** 10.1155/2016/5235269

**Published:** 2016-03-03

**Authors:** Bonaventura C. T. Mpondo, Daniel W. Gunda, Semvua B. Kilonzo, Erick Mgina

**Affiliations:** ^1^Department of Medicine, College of Health Sciences, The University of Dodoma, Dodoma, Tanzania; ^2^Department of Medicine, Catholic University of Health and Allied Sciences, Mwanza, Tanzania; ^3^Department of Data Management, National Institute of Medical Research, Tukuyu Unit, Mbeya, Tanzania

## Abstract

*Background*. Limited information exists on adults ≥50 years receiving HIV care in sub-Saharan Africa despite their increasing number. We aimed at studying immunologic and clinical responses to ART in this population.* Methods*. Data of patients who initiated HAART between 30th of June 2004 and 1st of May 2008 at Sekou Toure Care and Treatment Clinic were retrospectively analyzed. Date of ART initiation was used as a baseline and 48 months as a follow-up date. Immune recovery was defined as a CD4 count of ≥350 cells/mm^3^ at 48 months and late presentation as presentation with WHO stage 3 or 4 at clinic enrollment. Proportions of patients reaching this endpoint were compared between the two groups.* Results*. A total of 728 patients were included in our study; of these 73 (10.0%) were aged 50 years and above. Late presentation was more common in elderly patients than young patients (65.7% versus 56.1%, *P* = 0.12). Proportion of patients with CD4 count ≥350 (immune recovery) was higher in younger patients than in elderly patients, although this was not statistically significant (54.5% versus 44.9%, *P* = 0.2). Median absolute increase in CD4 at 48 months was higher in younger patients than in elderly patients (+241.5 cells/mm^3^ versus +146 cells/mm^3^, *P* = 0.007).* Conclusion*. Elderly HIV patients have higher rates of late presentation, with lower immune recovery. Strategies to increase HIV testing in this group are required for early diagnosis and treatment to improve outcomes.

## 1. Introduction

Globally the number of people living with HIV at advanced age is increasing [1]. In 2013, UNAIDS estimated that 4.2 million people living with HIV were aged 50 and above. In sub-Saharan Africa which harbors more than seventy percent of all HIV cases, it was estimated that there were 2.4 million people living with HIV at the age 50 and above [1]. The number is almost double that which was observed ten years ago. Projections show that this number will increase further and almost triple by the year 2030 [2]. Prolonged survival among HIV-infected patients following the use of ART explains such an increase [3]. Some studies have shown increased risk of HIV infection in this population [4]. However routine HIV testing is uncommon in this population, which makes them prone to late testing.

Highly active antiretroviral therapy (HAART) has been shown to be effective in reducing HIV viral load, thereby improving CD4 lymphopenia [5]. However the clinical and immunological responses in elderly patients treated with HAART have been mixed. There is controversy about the rates of CD4 response among elderly patients compared to younger patients. While some studies have shown lower CD4 recovery among older patients [6–8], others have not noted a significant difference in CD4 response between the two groups [9–11]. Many of the existing reports are from developed countries. Reports from sub-Saharan Africa which is the most affected region with HIV are scarce. This study aimed at assessing immunological and clinical responses following HAART use among elderly HIV-infected patients.

## 2. Materials and Methods

### 2.1. Study Design and Study Setting

Retrospective analysis of the data involving ART naïve patients initiating HAART between 30th of June 2004 and 1st of May 2008 at Sekou Toure Regional/Referral Hospital care and treatment clinic (CTC) in Mwanza, Northwestern Tanzania, was done.

HAART was defined as a combination of two nucleoside reverse transcriptase inhibitors plus a nonnucleoside reverse transcriptase inhibitor or a protease inhibitor with or without a pharmacological booster. The age cut-off value was set at 50 years (<50 and ≥50 years old at baseline) as used in other studies [6, 12, 13].

Collected variables included the age at ART initiation, sex, baseline CD4 cell count, WHO clinical stage at enrollment, occupation, marital status, type of first HAART, indications for ART, and time taken from ART initiation to ART change. Late diagnosis was defined as presentation with WHO clinical stage 3 or 4 at the time of enrollment. Immunological recovery was defined as CD4 cell count ≥350 cells/mm^3^ at 48 months of followup. Proportions of patients reaching this endpoint were compared between the two groups.

Data for this study were extracted from patient clinical files and data cleaning and descriptive data analysis was done using Stata software version 12.0 (Statcorp, Texas, USA). Comparisons between the two groups were computed using chi-squared test for categorical variables, nonparametric Wilcoxon rank-sum test for continuous variables, and Student's *t*-test to compare proportions between the groups and the comparison was considered significant at the *P* value less than 0.05.

## 3. Results

A total of 728 patients were included in our study; of these 73 (10.0%) were aged 50 years and above. The median age was 37 (IQR 32–42) among younger patients and 54 (IQR 52–57) among the elderly patients. Majority of participants were female in the younger group (69.9%) and male in the elderly group (58.9%). Comparisons of the baseline sociodemographic and clinical characteristics of the study participants are summarized in Table 1.

Late diagnosis was more common in elderly patients than young patients (65.7% versus 56.1%, *P* = 0.12). Median absolute increase in CD4 at 48 months was found to be significantly higher in younger patients than in their elderly counterpart (+241.5 cells/mm^3^ versus +146 cells/mm^3^, *P* = 0.007). We also found the proportion of patients with CD4 count ≥350 cells/ul at the time of followup (48 months) to be higher in the younger group (33.9% versus 30.1%, *P* = 0.2). The median absolute increase in body weight was found to be similar between the two groups (5.0 kg versus 5.0 kg, *P* = 0.52) as was the proportion of patients who had a change in ART regimen within the 48 months of followup (49.6% versus 38.4%, *P* = 0.06). The proportion of patients who had opportunistic infection at 48 months was similar between the groups (4.6% versus 4.1%, *P* = 0.81). Comparisons of immunological and clinical responses are summarized in Table 2.

## 4. Discussion

This study aimed at assessing the immunological response following the use of ART in HIV-infected patients aged 50 and above. Out of 728 study participants, 73 (10%) were aged 50 years and above. The age cut-off was set at 50 years as suggested in literature [12]. Elderly patients were diagnosed late compared to younger patients, although not significantly so. After 48 months of followup, the absolute median increase in CD4 count was significantly lower in elderly patients than in younger patients. More patients in the younger group had attained CD4 of ≥350 cells/ul months of followup. Proportions at 48 months of patients who changed ART regimen during followup were more in elderly patients than in younger patients, although not significantly so. However, the median weight gain was comparable between the two groups.

Studies comparing the efficacy of ART in elderly HIV-infected patients have been done elsewhere [10, 13–15]. Similar to our findings, other studies also found immunological response to be decreasing with increasing age [8, 16]. This can be explained by decreasing thymic volume with increasing age [17]. There is also a decline in production of naïve T cells with increasing age [18]. Old age is also associated with reduced memory T cell populations, impaired T cell functionality, and reduced number of properly functioning CD8 cytotoxic T cells [19].

The late testing in elderly patients has been described in other studies [15, 20, 21]. In our study, we also found that more elderly patients presented with HIV WHO clinical stages 3 and 4 diagnosed with advanced HIV (WHO clinical stage 3 or 4) compared to younger patients. However, the difference was not statistically significant. This is due to the fact that late presentation is also common in other age groups in sub-Saharan Africa as shown in other studies. Late diagnosis has been associated with impaired immune response [9], clinical progression, and increased risk of mortality [22]. More than half of the elderly patients (65.7%) in our study presented with advanced HIV. Other studies also found late presenters among elderly HIV-infected patients to be above 50% [12, 21, 22]. One of the reasons for late diagnosis could be the overlap between symptoms of HIV and those associated with ageing. Clinicians are also high unlikely to suspect HIV in this population, something that may delay the diagnosis.

Our study had several limitations. Analysis was based on data retrieved retrospectively from clinic database and patients' files; a lot of data was missing. Some important parameters such as treatment adherence are not routinely documented during clinic visit and could not be analyzed despite its importance. This study was based on a single clinic; the results may not necessarily be generalizable. In our study, there were few elderly patients in the study participants compared to younger ones. Our study included patients initiated on ART in June 2004 to May 2008. This long period could have contributed to the high rates of missing values. The missing data were equally distributed between groups and would not therefore be the reason for bias. To the best of our knowledge, this is the first study from Tanzania reporting on immunological responses following the use of ART among elderly HIV patients.

## 5. Conclusion

Elderly HIV-infected patients have an immunological response to ART. However their response is significantly lower than that observed in younger patients. The low immune response could be contributed by the late diagnosis of the disease which is more observed in elderly patients than younger patients in addition to the immunosenescence phenomena. We believe that our findings when coupled with the available guidelines for management of HIV patients have the potential to increase the rate of HIV testing with a subsequent early diagnosis and management. In addition, further studies are necessary to establish the long term outcome of these patients as well as the possibility of drug resistance.

## Figures and Tables

**Table 1:** Comparison of baseline sociodemographic and clinical characteristics among HIV-infected adults on ART attending care and treatment in Northwestern Tanzania, by age.

Variable	Age < 50 years	Age ≥ 50 y	*P* value
	(*n* = 654)	(*n* = 73)	
Median age years (IQR)	37 (32–42)	54 (52–57)	
Median baseline CD4	113 (43–197)	171.5 (72.5–245.5)	0.99
cells/ul (IQR)			
Sex			
Male	197 (30.1%)	43 (58.9%)	
Female	457 (69.9%)	30 (41.1%)	<0.0001
Occupation			0.99
Unemployed	26 (4.0%)	3 (4.1%)	
Employed	58 (8.9%)	2 (2.7%)	
Peasant	383 (58.6%)	45 (61.6%)	
Petty trader	182 (27.8%)	20 (27.4%)	
Marital status			
Single	105 (16.1%)	4 (5.5%)	
Married	346 (52.9%)	41 (56.2%)	
Divorced	90 (13.8%)	6 (8.2%)	
Widowed	104 (15.9%)	22 (30.1%)	0.004
Cohabiting	9 (1.4%)	0	
WHO clinical stage at			
enrollment			
Stages 1 and 2	287 (43.9%)	26 (34.3%)	
Stages 3 and 4	367 (56.1%)	48 (65.7%)	0.115
Indication for ART			
Adult CD4 < 200	525 (80.4%)	54 (74.0%)	
Adult WHO stage 3	88 (13.5%)	15 (20.6%)	
with CD4 < 350	12 (1.8%)	0	
Adult WHO stage 4	17 (2.6%)	1 (1.4%)	
Pregnancy	11 (1.7%)	3 (4.1%)	0.17
Body weight at			
enrollment (IQR)	54 (48–60)	55 (50–60)	0.87

**Table 2:** Clinical and immunological outcomes following the use of ART among HIV-infected adults attending care and treatment centre in Northwestern Tanzania, by age and sex.

Variable	Sex			
	Male *n* (%)		Female *n* (%)	
	15–49	≥50	15–49	≥50
CD4 counts of cell/ul				
≤350	68 (34.3)	15 (34.9)	117 (25.6)	12 (40.0)
>350	53 (26.8)	12 (27.9)	169 (37.0)	10 (33.3)
Missing	77 (38.9)	16 (37.2)	171 (37.2)	8 (26.7)
	0.98		0.2	
Change in ART				
Yes	87 (43.9)	29 (67.4)	238 (52.1)	16 (53.3)
No	109 (55.1)	14 (52.6)	216 (47.3)	14 (46.7)
Missing	2 (1.0)	0 (0.0)	3 (0.7)	0 (0.0)
	0.02		0.9	
Opportunistic infection at follow-up				
Yes	15 (7.6)	7 (16.3)	23 (5.0)	2 (6.7)
No	183 (92.4)	36 (83.7)	434 (95.0)	28 (93.3)
Missing	0 (0.0)	0 (0.0)	0 (0.0)	0 (0.0)
	0.07		0.69	
Reported admission at follow-up				
Yes	27 (13.6)	6 (14.0)	72 (15.8)	1 (3.3)
No	171 (86.4)	37 (86.0)	385 (84.2)	29 (96.7)
Missing	0 (0.0)	0 (0.0)	0 (0.0)	0 (0.0)
	0.96		0.06	
Other comedications at follow-up				
Yes	179 (90.4)	41 (95.4)	421 (92.1)	30 (100.0)
No	16 (8.1)	2 (4.6)	33 (7.2)	0 (0.0)
Missing	3 (1.5)	0 (0.0)	3 (0.7)	0 (0.0)
	0.52		0.28	
